# Ultrasound real-time elastography can predict malignancy in BI-RADS^®^-US 3 lesions

**DOI:** 10.1186/1471-2407-13-159

**Published:** 2013-03-27

**Authors:** Sebastian Wojcinski, Esther Boehme, André Farrokh, Philipp Soergel, Friedrich Degenhardt, Peter Hillemanns

**Affiliations:** 1 Hannover Medical School, Department of OB/GYN, OE 6410, Carl-Neuberg-Straße 1, Hannover, 30625, Germany; 2 Franziskus Hospital, Department of OB/GYN, Bielefeld, Germany

**Keywords:** Sonoelastography, Breast lesions, BI-RADS-US 3, Diagnostic accuracy, Breast cancer, Breast imaging

## Abstract

**Background:**

Lesions of the breast that are classified BI-RADS^®^-US 3 by ultrasound are probably benign and observation is recommended, although malignancy may occasionally occur.

In our study, we focus exclusively on BI-RADS^®^-US 3 lesions and hypothesize that sonoelastography as an adjunct to conventional ultrasound can identify a high-risk-group and a low-risk-group within these patients.

**Methods:**

A group of 177 breast lesions that were classified BI-RADS^®^-US 3 were additionally examined with real-time sonoelastography. Elastograms were evaluated according to the Tsukuba Elasticity Score. Pretest and posttest probability of disease (POD), sensitivity (SE), specificity (SP), positive (PPV) and negative predictive values (NPV) and likelihood-ratios (LR) were calculated. Furthermore, we analyzed the false-negative and false-positive cases and performed a model calculation to determine how elastography could affect the proceedings in population screening.

**Results:**

In our collection of BI-RADS^®^-US 3 cases there were 169 benign and eight malignant lesions. The pretest POD was 4.5% (95% confidence interval (CI): 2.1–9.0). In patients with a suspicious elastogram (high-risk group), the posttest POD was significantly higher (13.2%, p = 0.041) and the positive LR was 3.2 (95% CI: 1.7–5.9). With a benign elastogram (low-risk group), the posttest POD decreased to 2.2%. SE, SP, PPV and NPV for sonoelastography in BI-RADS^®^-US 3 lesions were 62.5% (95% CI: 25.9–89.8), 80.5% (95% CI: 73.5–86.0), 13.2% (95% CI: 5.0–28.9) and 97.8% (95% CI: 93.3–99.4), respectively.

**Conclusions:**

Sonoelastography yields additional diagnostic information in the evaluation of BI-RADS^®^-US 3 lesions of the breast. The examiner can identify a low-risk group that can be vigilantly observed and a high-risk group that should receive immediate biopsy due to an elevated breast cancer risk.

## Background

More than one million women are newly diagnosed with breast cancer each year [1]. Industrial nations with the highest cancer rates include the U.S.A, Italy, Australia, Germany, the Netherlands, Canada and France [2]. In 2000, the last year for which global data exists, approximately 400,000 women died from breast cancer, representing 1.6 per cent of all female deaths [3]. The early detection of breast cancer has moved into the very focus of primary health care. There is sound evidence that the recent decline in cancer mortality observed in several countries is due to early detection to a significant extent [4]. The responsibility for this success includes improvements in imaging technologies, but is also due to a higher degree of health awareness and educational programs. Nevertheless, a significant number of false-positive and false-negative findings still occur [5]. The consequence of a false-positive result in diagnostic imaging is the performance of an unnecessary biopsy. A false-negative result has an even more serious implication as the diagnosis of malignancy is delayed, with a potentially worse clinical outcome for the patient. In order to prevent excessive biopsies on the one hand and, in particular, to guarantee the highest level of patient safety on the other hand, diagnostic methods should be continuously refined.

Today, ultrasound (US) plays a decisive role in the diagnostic pathways, with high sensitivity and specificity [6]. Despite technical advances, the most important step in bringing breast US to its current position was the introduction of the standardized BI-RADS^®^-US-classification system by the American College of Radiology (ACR) [7]. The ACR BI-RADS^®^–US lexicon provides various categories with predefined terminology to describe the dominant features of breast lesions accurately. According to the ACR, each lesion should be assigned a BI-RADS^®^-US category ranging from BI-RADS^®^-US 0 to BI-RADS^®^-US 6 at the end of the diagnostic procedure [7]. The distinct BI-RADS^®^-US classification also implies what further clinical action should be taken: BI-RADS^®^-US 4 lesions are possibly malignant and BI-RADS^®^-US 5 lesions are probably malignant. Therefore, the appropriate consequence is a biopsy, usually under US guidance. Malignancy practically never occurs in BI-RADS^®^-US 2 lesions, which are defined as benign findings.

To our understanding, the group of BI-RADS^®^-US 3 lesions remains a critical category. These findings are probably benign and short-term follow-ups are recommended. Nevertheless, malignancy is eventually diagnosed in about 3% of these lesions, resulting in a delayed diagnosis of cancer in a considerable number of patients [8]. Therefore, a suitable predictor for malignancy in BI-RADS-^®^-US 3 lesions would be beneficial and of clinical relevance.

**Figure 1:**
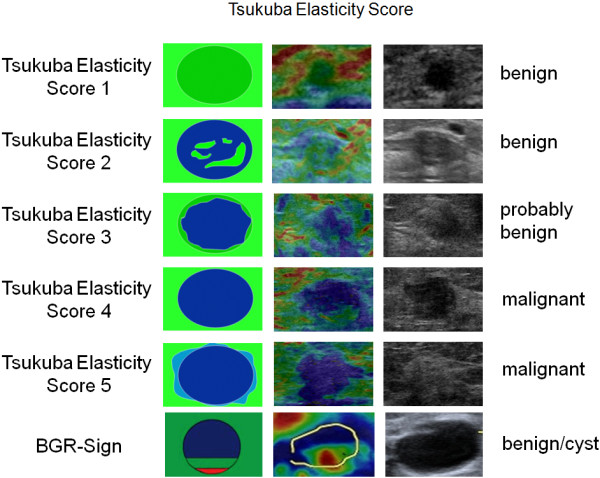
**Tsukuba Elasticity Score (TES).** Schematic view, sonoelastography and B-mode ultrasound of lesions categorized as TES 1–5 or BGR-sign.

Nowadays, newly developed US technologies may allow a better differentiation of benign and malignant masses [9]. The idea behind real-time tissue elastography (sonoelastography) is that a region of interest is subjected to a compression force (stress) and the resulting strain (displacement) within the tissue is assessed [Figure 1]. The most common way to apply stress is to compress the tissue with the ultrasound probe (hand-held sonoelastography). Another recently developed method is shear wave elastography, which uses an acoustic push pulse to induce an elastic shear wave that propagates through the tissue. The velocity of the shear wave is measured by detection pulses and provides a quantitative measurement of tissue stiffness [10].

Our study was based on hand-held sonoelastography. In a recent meta-analysis regarding all BI-RADS^®^-US categories, this non-invasive ultrasound-based method demonstrated a good sensitivity of 79% and an excellent specificity of 88% compared to conventional B-mode US (96% and 70%, respectively) [11]. Furthermore, when sonoelastography is applied to a predefined group of lesions, a diagnostic advantage may be achieved. This has been demonstrated in a subgroup analysis of BI-RADS^®^-US 3 lesions, where the probability of disease (POD) significantly increased from 8.3% to 45.5% with a suspicious elastogram [6].

Therefore, we designed our prospective study to concentrate exclusively on BI-RADS^®^-US 3 lesions, and scrutinize whether sonoelastography can truly filter out malignancy in this group.

Our hypothesis is that elastography can identify a high-risk group (prevalence of malignancy significantly increased) and a low-risk group (prevalence of malignancy significantly decreased) within the cohort of patients with a BI-RADS^®^-US 3 lesion (overall prevalence of malignancy about 3%).

## Methods

### General design

Our study was carried out at the Breast Cancer Center of Franziskus Hospital in Bielefeld, Germany. The study cohort was selected from patients who were referred to our clinic due to specific diagnostic queries. Patients with a sonographically visible lesion categorized as BI-RADS^®^-US 3 were regarded as being suitable for our study. Patients with a current treatment of breast cancer, skin disorders, inflammatory conditions of the breast, current pregnancy or lactation or psychiatric problems were excluded. Ultrasound examinations and sonoelastography were not performed under study conditions but for diagnostic purposes within the routine practice of our breast cancer center. As we were using a standard of care clinical protocol, the responsible ethics committee did not require additional approval for the non-interventional design of our prospective case study (Hannover Medical School, Ethics committee, Study-ID 1414–2012).

### Ultrasound examinations

Ultrasound examinations were performed by the author SW, a DEGUM (Deutsche Gesellschaft für Ultraschall in der Medizin, German society for ultrasound in medicine) level II certified senior consultant in gynecology with 7 years’ experience in breast US [12]. For sonography, two high-end ultrasound scanners were used:

1. The Siemens ACUSON S2000™ ultrasound system (Siemens Medical Solutions, Inc., Mountain View, CA, USA) equipped with the 18 L6 HD linear transducer (5.5–18 MHz, 5.7 cm)

2. The Hitachi HI VISION Avius^®^ ultrasound system (Hitachi Medical Corporation, Inc., Tokyo, Japan) equipped with the EUP L74M linear transducer (5–13 MHz, 5.0 cm) or the EUP-L65 linear probe (6–14 MHz, 3.8 cm)

One-hundred and fifty-three lesions were scanned with the ACUSON S2000™ and 24 lesions were scanned with the Hitachi HI VISION Avius^®^.

**Figure 2:**
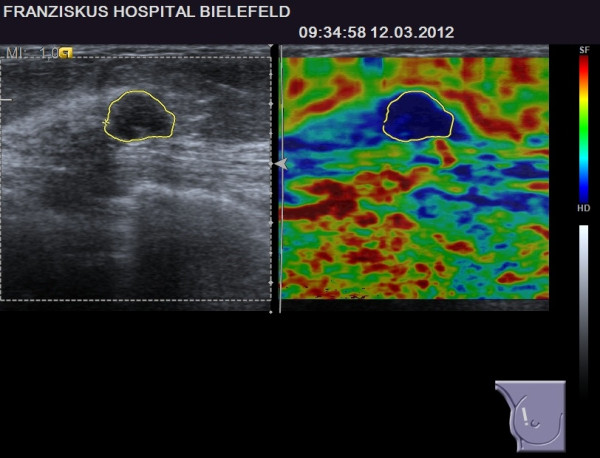
**BI-RADS^®^-US 3 lesion with a suspicious elastogram, TES 4.** The lesion was revealed to be malignant (true positive case).

**Figure 3:**
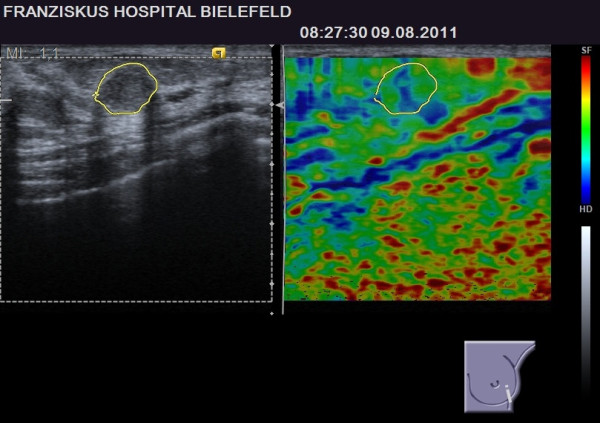
**BI-RADS^®^-US 3 lesion with a benign elastogram, TES 2.** The lesion was revealed to be malignant (false negative case).

**Figure 4:**
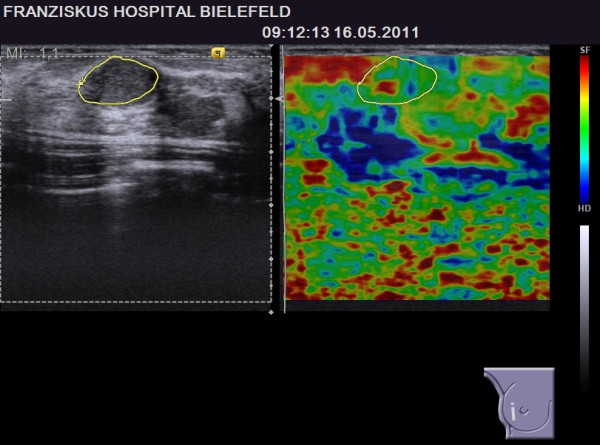
**BI-RADS^®^-US 3 lesion with a benign elastogram, TES 2.** The lesion was revealed to be benign (true negative case).

**Figure 5:**
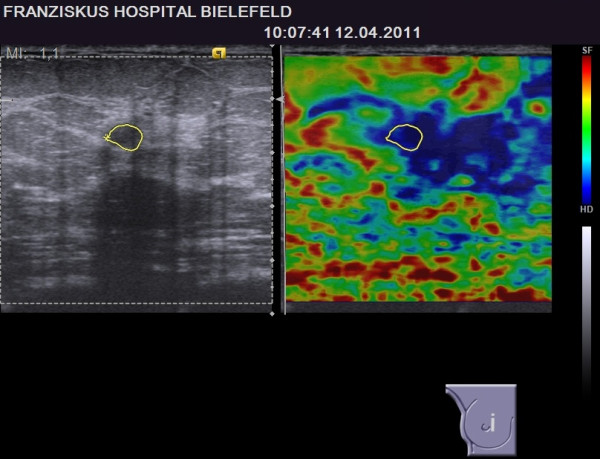
**BI-RADS^®^-US 3 lesion with a suspicious elastogram, TES 5.** The lesion was revealed to be benign (false positive case).

All patients received bilateral whole breast ultrasound. B-mode pictures of the lesion were obtained and documented in two planes. As previously reported, the orientation of the probe does not influence the elastographic score [13]. Therefore, the elastograms were taken either in the sagittal or horizontal orientation. In the most common mode for sonoelastography, hard tissue appears blue to turquoise and soft tissue appears red to green on a continuous scale ranging from red through yellow, green and turquoise to blue [Figures 1, 2, 3, 4 and 5].

According to the national regulating authority statutes, breast US systems have to fulfill basic technical requirements and undergo regular quality control measures [14]. These standards applied to the equipment used in our study.

Following the ultrasound examination, further diagnostic steps (i.e. magnetic resonance imaging, mammography, core needle biopsy, open biopsy or follow-up examinations) were conducted whenever necessary. Finally, we used the following means to define the benign or malignant nature of the lesions:

57 lesions (32.2%) were followed up after 6 months and/or additionally examined by other imaging methods and finally staged down to BI-RADS^®^ 2 and consequently categorized as certainly benign. In 120 cases (67.8%) it was considered necessary to perform a fine needle aspiration, an open biopsy or a core needle biopsy at the time of the first consultation or during one of the follow-up visits.

### Image interpretation and data management

The elastograms were evaluated using the Tsukuba Elasticity Score (TES), also known as the Itoh-Score or Elasto-Score, which is a 5-point strain partly corresponding to the BI-RADS classification [Figure 1]. The cut-off point for malignancy is between TES 3 and TES 4 [15]. Therefore, lesions that are categorized as TES 1, 2 or 3 are considered probably benign, and lesions categorized as TES 4 and 5 are suspicious for cancer.

Due to technical reasons, liquid cysts are regularly associated with the BGR-sign (blue-green-red-sign, a color pattern with blue in the top area of the lesion, green in the middle and red at the bottom that particularly applies to the Hitachi system) or with a color distribution resembling a “bull’s-eye” (which particularly applies to the Siemens system). Lesions displaying these color distributions are defined as probably benign, as this phenomenon is regularly and exclusively observed in cysts [16,17].

### Statistics

Microsoft^®^ Office Excel^®^ 2003 (Microsoft Corporation) was used for data collection.

Statistical analysis was performed using MedCalc^®^ 11.6 statistical software (MedCalc Software bvba, Belgium). In the primary analysis, we calculated the pretest probability of disease (POD) as well as the posttest POD in the high-risk group (suspicious elastogram) and in the low-risk group (benign elastogram). Pairwise comparisons were performed using the Z-test. Furthermore, we calculated the positive likelihood ratio (high-risk group) and the negative likelihood ratio (low-risk group). If the lower boundary was greater than one for the high-risk group and if the upper boundary was less than one for the low-risk group the null hypotheses could be rejected. Secondarily, we calculated sensitivity, specificity and predictive values based on the Bayesian theorem. For the calculation of 95% confidence levels we used Newcombe intervals with continuity correction [18]. Normality was tested with the Agostino-Pearson-test. The Student’s *t*-test was used for numerical data and comparison of means when normality holds. For distributions far from normal, the Mann–Whitney *U* test was used. Statistical significance was assumed at p < 0.05 for all tests.

Furthermore, we performed a model calculation to determine how elastography could affect the proceedings in population screening with a given prevalence of BI-RADS^®^-US 3 lesions. For this purpose, we compared data from the literature concerning the biopsy rate in BI-RADS^®^-US 3 lesions with the estimated proportion of BI-RADS^®^-US 3 lesions that would be biopsied after an additional elastogram by simple percentage calculation.

## Results

Data were collected between June 2009 and April 2012. The study patients had an average age of 48.0 ± 14.3 years, ranging from 16 to 79 years. In total, data from 177 BI-RADS^®^-US 3 lesions in 153 patients were collected. The average diameter of the lesions was 14 ± 7 mm (range 4 to 46 mm). Finally, 169 lesions (95.5%) were identified as benign and 8 lesions (4.5%, 95% CI: 2.1-9.0) were found to be malignant.

### Primary analysis: probability of disease and likelihood ratios

The pretest POD was 4.5% (95% CI: 2.1–9.0) and rose significantly to 13.2% (95% CI: 5.0–28.9) with a suspicious elastogram in the high-risk group (p = 0.041). With a benign elastogram, the posttest POD decreased to 2.2% (95% CI: 0.6–6.7) in the low-risk group (p = n.s.) [Table 1].

**Table 1:** Primary outcome analysis

**Effect of sonoelastography on the probability of disease**		
	**TES 1–3 or BGR-sign (test negative, benign elastogram, low-risk group)**	**TES 4–5 (test positive, suspicious elastogram, high-risk group)**
pretest POD	4.5% ^a, b^ (2.1-9.0)	
posttest POD	2.2% ^b, c^ (0.6-6.7)	13.2% ^a, c^ (5.0-28.9)
Likelihood ratio	0.5 (0.2-1.1)	3.2 (1.7-5.9)

Comparison of pretest- and posttest-POD and likelihood ratios.

95% confidence interval in brackets.

POD = probability of disease; TES = Tsukuba elasticity score; BGR = blue-green-red.

^a^ p = 0.041 (comparison of POD before test and POD after positive test).

^b^ p = 0.268 (comparison of POD before test and POD after negative test).

^c^ p = 0.004 (comparison of POD after negative test and POD after positive test).

Accordingly, the negative likelihood ratio was 0.5 (95% CI: 0.2–1.1). Therefore, the upper boundary for the low-risk group marginally failed to be smaller than one, and for this group the null hypothesis could not be rejected.

Moreover, for the high-risk group, the positive likelihood ratio was 3.2 (95% CI: 1.7–5.9). In this group the lower boundary was greater than one and, consequently, we could reject the null hypothesis.

### Sensitivity, specificity, predictive values and prevalence of malignancy

The sensitivity of the sonoelastography was 62.5% (95% CI: 25.9–89.8), which means that sonoelastography could recognize approximately two out of three malignancies in the group of BI-RADS^®^-US 3 lesions. The specificity was 80.5% (95% CI: 73.5–86.0), and the positive and negative predictive values were found to be 13.2% (95% CI: 5.0–28.9) and 97.8% (95% CI: 93.3–99.4), respectively [Table 2].

**Table 2:** **Sensitivity, specificity and predictive values for sonoelastography in BI-RADS^®^-US 3 lesions**

**Sensitivity, specificity and predictive values for sonoelastography**	
Prevalence of malignancy in BI-RADS^®^-US 3	4.5% (2.1-9.0)
Sensitivity	62.5% (25.9-89.8)
Specificity	80.5% (73.5-86.0)
Positive predictive value	13.2% (5.0-28.9)
Negative predictive value	97.8% (93.3-99.4)

95% confidence intervals in brackets.

### Descriptive analysis of the malignant lesions

The prevalence of malignancy was 4.5% (95% CI: 2.1–9.0) for the BI-RADS^®^-US 3 lesions in our study, as we eventually detected eight breast cancer cases. Out of these eight lesions, none was categorized TES 1, two lesions were categorized TES 2, one lesion was categorized TES 3 and five lesions were correctly categorized TES 4 (n = 4) or TES 5 (n = 1) [Table 3].

**Table 3:** Prevalence of malignant lesions within the different elastography scores

**Prevalence of malignancy according to the Tsukuba Elasticity Score**		
**TES**	**n**	**Prevalence of malignant lesions**
1	7	0% (0.0-43.9)
2	82	2.4% (0.4-9.4)
3	46	2.2% (0.1-13.0)
4	29	13.8% (4.5-32.6)
5	9	11.1% (0.6-49.3)
BGR-sign	4	0% (0.0-60.4)

95% confidence interval in brackets. TES = Tsukuba elasticity score; BGR = blue-green-red.

The characteristics of the eight breast cancer cases are shown in Table 4.

**Table 4:** Characteristics of the breast cancer patients in our study sorted by the results from the elastogram (comparison of true positives and false negatives)

**Characteristics of the breast cancer patients**							
**Age**	**Tumor type**	**Longest axis on histology (mm)**	**Longest axis on US (mm)**	**Distance from skin on US (mm)**	**TES**	**BI-RADS on MG**	**Time since last MG (months)**
True positive cases (suspicious elastogram in breast cancer patients)							
77	IDC	21	17	5	4	3	0
73	IDC	15	22	5	4	3	14
77	DCIS	10	14	2	4	0	0
39	DCIS	5	12	6	4	n.a.^1^	n.a.^1^
58	IDC	12	12	6	5	2	12
False negative cases (benign elastogram in breast cancer patients)							
71	IDC	8	8	12	2	4	0
48	IMC	8	9	4	2	2	0
71	IDC	11	12	18	3	3	0

US = ultrasound; TES = Tsukuba elasticity score; IDC = invasive ductal carcinoma; DCIS = ductal carcinoma in situ; IMC = invasive mucinous carcinoma; MG = mammography; n.a. = not available.

^1^not available: The patient presented with a palpable lesion that was exclusively diagnosed with ultrasound as the patient was less than 40 years old.

Seven patients presented with a mammogram, but there was only one patient with a suspicious mammogram (BI-RADS 4) and one patient with a mammogram that recommended additional imaging evaluation (BI-RADS 0). The other breast cancer patients (n = 5) had benign or probably benign mammograms (BI-RADS 2 or 3).

With eight patients, the breast cancer group is too small to permit statistical comparison of true-positive (suspicious elastogram) and false-negative (benign elastogram) cases, but there was a tendency for smaller lesions located deeper in the breast in the subgroup of false-negative elastograms [Table 4]. Examples of a true-positive and a false-negative breast cancer case are given in Figures 2 and 3.

### Analysis of the benign lesions

Focusing on the 169 benign lesions, 136 elastograms were benign and 33 elastograms were suspicious. Therefore, elastography showed a false-positive result in approximately one out of five benign BI-RADS^®^-US 3 lesions.

Comparing these true-negative and false-positive cases, there was no difference regarding the size of the lesions or their distance from the skin. Regarding age, women with a suspicious elastogram (false-positive cases) were significantly older than women with a benign elastogram (true-negative cases) (53.1 versus 45.9 years, p = 0.008) [Table 5]. Examples of a true-negative and a false-positive result are given in Figures 4 and 5.

**Table 5:** Characteristics of women with benign lesions sorted by the results from the elastogram (comparison of true positives and false negatives)

**Characteristics of women with benign lesions**			
	**True negative cases (benign elastogram in benign lesions)**	**False positive cases (suspicious elastogram in benign lesions)**	**p**
N	136	33	
Age (years; mean ± SD)	45.9 ± 14.0	53.1 ± 10.7	0.008^1^
Distance from skin in US (mm; mean ± SD)	6.6 ± 3.8	7.3 ± 3.3	n.s.
			(0.158)^2^
Longest axis in US (mm; mean ± SD)	13.7 ± 6.1	14.2 ± 9.5	n.s.
			(0.361)^2^
US-platform	S: 84.6%	S: 97.0%	n.s.
	H: 15.4%	H: 3.0%	(0.057)^3^

US = ultrasound; SD = standard deviation.

^1^ Student’s *t*-test.

^2^ Mann–Whitney *U* test.

^3^ Z-Test.

### Model calculation

Based on our findings, we performed a model calculation to estimate how elastography could affect the proceedings in screening populations. As demonstrated in the ACRIN 6666 trial, BI-RADS^®^-US 3 lesions occur in about 19.5% of high-risk women and at least 16.6% of BI-RADS^®^-US 3 patients can be expected to be ultimately biopsied as the lesions are upgraded in the follow-up examinations [19]. This means that about 3.2% of all screened women usually need a biopsy due to a BI-RADS^®^-US 3 lesion. In our study, 21.5% of BI-RADS^®^-US 3 lesions had a suspicious elastogram and immediate biopsy was recommended. Consequently, focusing on patients with BI-RADS^®^-US 3 lesions, the biopsy rate would increase from 16.6% (management without elastography) to 21.5% (indication to biopsy based on the elastogram). Focusing on the screening population, in which BI-RADS^®^-US 3 lesions only occur in about 20% of the women, the overall biopsy rate of 3.2% (without elastography) would increase to 4.2% (with elastography). However, this would allow the detection of 62.5% of cancers directly during the first consultation, instead of performing the biopsy many months or even years later after the tumor progresses in the follow-up examinations.

## Discussion

### Diagnostic performance of sonoelastography in BI-RADS^®^-US 3 lesions

Compared to mammography and conventional ultrasound, sonoelastography has previously demonstrated an excellent diagnostic performance in the evaluation of breast lesions [6]. Nevertheless, there are plenty of clinical situations where such additional information is not imperative. Patients almost never receive a single imaging technique alone (i.e. conventional ultrasound, mammography or magnetic resonance imaging), but usually undergo several different examinations before receiving a final diagnosis. Therefore, in a realistic setting, sonoelastography is not applied as a single method but is used in addition to other examinations in “heavily-pre-diagnosed patients”.

An elastogram, for example, might be capable of identifying a breast lesion as highly suggestive of malignancy, but if this lesion was already suspicious on the mammogram (i.e. BI-RADS^®^ 5) and/or in the conventional ultrasound (i.e. BI-RADS^®^-US 5), this additional information would not have an effect on the management of the patient.

Therefore, we need to concentrate on patients and breast lesions where the appropriate action is as yet unclear and a more advanced assessment is needed. This is the rationale for our focus on patients with BI-RADS^®^-US 3 lesions. The risk for malignancy is relatively low in this category, but can reach 3% or even more in distinct patient populations [7,8,20]. In our study, the prevalence of breast cancer was moderately higher (4.5%), but the 95% CI (2.1-9.0) suggests that our population shows an acceptable distribution of benign and malignant cases and can be regarded as representative for other populations. Sonoelastography demonstrated a helpful discrimination of patients into a low-risk group (posttest POD 2.2%) or a high-risk group with a significantly increased posttest POD of 13.2%. In addition, most of the breast cancer patients in our study had a benign or probably benign mammogram (i.e. BI-RADS^®^ 2 or 3). Accordingly, further diagnostic steps were initially not indicated and the elastogram alone led to the diagnosis of cancer.

This is why we recommend the application of sonoelastography to BI-RADS^®^-US 3 lesions. The decision of whether to take a histological specimen or merely perform follow-up examinations in a patient can then be based on the results from the elastogram.

### Can we detect more cancers even as we reduce the number of biopsies?

A missed cancer or a delayed diagnosis of cancer may have a decisive impact on the survival of a patient. Therefore, a perfectly arranged diagnostic setting is crucial for our patients.

Although BI-RADS^®^-US 3 lesions are probably benign, there is still a substantial risk of malignancy for the individual patient. As demonstrated by our results, sonoelastography has the power to identify a high-risk group with a three-fold increased risk of malignancy as well as a low-risk group with a decreased risk of malignancy. Nevertheless it has to be discussed whether this procedure can be transferred into our daily clinical routine. When performing breast diagnostics, there are two major aims: On the one hand, we intend to increase the number of correctly and early identified breast cancers (and consequently avoid missed malignancies and delayed diagnoses of cancer); on the other hand we try to reduce the number of unnecessary biopsies. In fact, both aims often behave reciprocally with each other. Nevertheless, our investigations should be aimed at developing methods that have a significant effect on diagnostic accuracy, but which only cause a slight increase in the biopsy rate.

Concerning BI-RADS^®^-US 3 lesions, performing short-term follow-up examinations has been suggested [7]. However, at least 16.6% of these patients usually need a biopsy during one of these follow-ups, as the tumor progresses or changes its morphology [19]. Furthermore, we experience an increase of biopsies in BI-RADS^®^-US 3 lesions, as patients often demand a histological confirmation rather than repeated examinations. As a consequence, many patients with a BI-RADS^®^-US 3 lesion are still biopsied today despite all of the recommendations.

Based upon this experience, sonoelastography would only slightly increase the number of biopsies if this technology were to be used as an adjunct to conventional ultrasound. We estimate that, in the group of BI-RADS^®^-US 3 lesions, the biopsy rate would increase by 4.9% (from 16.6% to 21.5%), but as BI-RADS^®^-US 3 lesions only occur in every fifth woman, the overall biopsy rate in the entire population would only rise by 1%. What is more, two thirds of all cancers in this group could be diagnosed immediately at the time of the first examination. To our way of thinking, the relationship between the only slightly increased biopsy rate and the positive predictive value of sonoelastography is definitely favorable. So far, elastography has not shown an ability to decrease the biopsy rate, but patient safety could be much higher with this technology.

### Why does sonoelastography fail in some cancers?

In our study, five out of eight breast cancers were correctly classified as suspicious on the elastogram, but there were also three false-negative results.

In 2006, Thomas et al. published data on 108 breast tumor patients [21]. The authors concluded that there may be limitations to sonoelastography for lesions that are extensive, inhomogeneous, located deep in the breast or related to rare histological types [21]. In our study, false-negative cases showed a tendency to be located deeper in the breast, which matches the experiences of Thomas et al.

In 2010, Farrokh et al. investigated limitations of the method in 222 breast lesions and put forward the idea that the accuracy of sonoelastography may be impaired in mucinous carcinoma of the breast [22]. Interestingly, our only case of a mucinous carcinoma also exhibited a benign elastogram. Mucinous carcinomas show a characteristic pattern of solid portions consisting of tumor cells and relatively soft areas consisting of liquid mucus. This growth pattern can be expected to have an effect on the physical properties of the tumor, i.e. these tumors can be expected to appear much softer on the elastogram than entirely solid tumors. This is further consistent with the idea that the elastogram is impaired in invasive mucinous carcinoma. Furthermore, Farrokh et al. experienced that the accuracy of sonoelastography decreased when the tumor diameter was larger than 20 mm [22].We have no data for this latter hypothesis, as none of the tumors in our study was significantly larger than 20 mm.

### Why does sonoelastography show suspicious results in benign lesions?

Tissue stiffens as malignant tumors form and grow, but this effect may be also observed in benign lesions. Therefore, the positive predictive value of sonoelastography remains low and there are a number of false-positive results, especially in populations with a low prevalence of malignity. Nevertheless, we identified one major variable that was strongly associated with false-positive elastograms. Women with a benign lesion and a suspicious elastogram (false-positive group) were significantly older than women with a benign lesion and a benign elastogram (true-negative group). Our finding can be explained by the fundamental principle of hand-held sonoelastography: This technology allows an estimation of the elasticity of the tissue by comparing different structures within the region of interest before and after applying stress. As a result, the mechanical properties of the surrounding tissue have a decisive impact on the appearance of the lesion on the elastogram. Usually, benign lesions appear soft on sonoelastography, but a benign lesion may also appear relatively hard if the adjacent tissue is relatively soft. This model of an “elastographic contrast” has been described before [23]. Focusing on benign lesions, this “elastographic contrast” seems to work better in relatively hard (meaning dense) breast tissue. Consequently, the correct classification of a benign lesion in a less dense breast seems to be more difficult with sonoelastography. Finally, breast density is strongly correlated with the patient’s age. As the breast tissue becomes less dense with age, the “elastographic contrast” diminishes for benign lesions and the false-positive rate rises, as demonstrated by our results. Hence, we support the concept of an “elastographic contrast”.

### Implications of the Tsukuba elasticity score

The so-called Tsukuba Elasticity Score, which is commonly used for sonoelastography, has never been prospectively validated in larger populations. Therefore, we lack final data demonstrating that the performance characteristics of the TES are suitable for the evaluation of elastograms. Nevertheless, the TES illustrated reproducible results in multiple studies and a recent meta-analysis [6,11,21]. The cut-off point to discriminate benign and malignant lesions is established between TES 3 and 4. In our study, the risk for malignancy was actually low for TES 1, 2 and 3 (0%, 2.4% and 2.2%, respectively) and considerably higher for TES 4 and 5 (13.8% and 11.1%, respectively). Hence, we consider this gradation as reliable. Nevertheless, up to 13% malignancy has been reported for TES 3 lesions in some studies [15]. Therefore, as long as the final validation of the score is not provided, each lesion must be carefully interpreted in the clinical context. An alternative for the TES could be an analysis of the strain-ratio (fat-lesion-ratio). This method has also shown good performance in the evaluation of breast lesions [24]. However, we decided to use the TES, as this score was the first classification system for elastograms in the literature, there is the most experience with this score, as the majority of cases in the literature were analyzed with the TES.

### The application of different ultrasound systems

For the examinations, we used two different ultrasound systems that were available in our breast cancer center. As the applied elastography modules are based on similar technologies, we presume that there are no differences. However, our study was not intended or empowered to explore differences between the two ultrasound systems. So far, there are no larger studies comparing different sonoelastography systems, but smaller cases series suggest that there is probably no major influence on the elastographic score [25].

### Limitations of the study

The main limitation of our study is that analysis of elastograms is, to a certain degree, observer-dependent, as it is based on image interpretation. It should be noted that conventional breast ultrasound and evaluation of B-mode images is also observer-dependent and a matter of subjective interpretation. Therefore, this limitation is inherent in sonoelastography per se and conventional B-mode ultrasound as well. So far, computer-aided diagnosis has shown little benefit for the analysis of ultrasound elasticity images and, in our view, visual assessment of elastograms by the examiner remains the gold-standard [26].

Another limitation is the lack of multiple observers and multiple study sites. All examinations were carried out by the same examiner, who had intensive training and many years’ experience with sonoelastography. To evaluate the performance under realistic conditions, the study concept should be repeated with multiple observers with different levels of experience. The outcome bias was minimized in our study setting, as the examiner did not know the final nature of the lesion at the time of the elastographic assessment. Furthermore, the B-mode images yielded no additional information, as all lesions were categorized BI-RADS^®^-US 3 and appeared probably benign.

Finally, to prove our concept, we see an imperative need for a prospective, multicenter trial to fully evaluate breast sonoelastography in a standard clinical setting. Furthermore, studies focusing on other risk groups (i.e. BI-RADS^®^ 4a, 4b and 4c) would be beneficial.

## Conclusions

Sonoelastography yields additional diagnostic information in the evaluation of BI-RADS^®^-US 3 lesions of the breast and enables the examiner to identify a low-risk group that can be watchfully observed as well as a high-risk group that should receive immediate biopsy due to an elevated breast cancer risk.

## Competing interests

The authors declare that they have no competing interests.

## Authors’ contributions

SW and AF contributed to the conception and design of the study and SW performed the ultrasound examinations. EB performed data collection. SW and EB contributed to the statistical analysis and the writing of the manuscript. PH and PS conducted final reviews of the database and the manuscript and FD provided additional methodological advice. All authors read and approved the final manuscript.

## Pre-publication history

The pre-publication history for this paper can be accessed here:

http://www.biomedcentral.com/1471-2407/13/159/prepub
